# Bari Shoulder Telemedicine Examination Protocol (B-STEP): A Standard Protocol for Personalized Remote Shoulder Examination

**DOI:** 10.3390/jpm13071159

**Published:** 2023-07-20

**Authors:** Lorenzo Moretti, Davide Bizzoca, Giacomo Farì, Alessandro Caricato, Francesco Angiulli, Giuseppe Danilo Cassano, Giuseppe Solarino, Biagio Moretti

**Affiliations:** 1 Orthopaedic and Trauma Unit, AOUC Policlinico di Bari, University of Bari “Aldo Moro”, Piazza Giulio Cesare 11, 70124 Bari, Italy; 2 Physical Medicine and Rehabilitation Unit, University of Bari “Aldo Moro”, 70124 Bari, Italy

**Keywords:** telemedicine, shoulder, examination protocol

## Abstract

The COVID-19 pandemic drastically changed many aspects of the traditional functioning of health systems all around the world. In Italy, as reported by the CIO, compared to the previous year, there was a significant reduction in 2020 in overall outpatient activities by up to 75%. These data support the need for telemedicine, which represents a current challenge and can no longer be postponed in the future. This study aims to elaborate on a possible model for remote shoulder examination based on traditional tests to improve the quality of telemedicine in orthopedic and rehabilitation. Between May 2020 and November 2020, ten orthopedic surgeons individually examined six patients with a known shoulder disorder, both in hospital and via webcam according to the previously shared protocol (B-STEP). According to the 10 observers, completing 100% of the ASES score and at least 87.5% of the Constant score is possible. Shoulder ROM and many specific tests are also reproducible via webcam, but with less sensitivity, according to the subjective opinion of observers. The B-STEP is a useful protocol for the standardization of the objective examination of the shoulder via webcam. Further studies are necessary to determine if the B-STEP protocol is useful for diagnosing pathology in unknown patients and evaluating its sensitivity and specificity for each pathology.

## 1. Introduction

The COVID-19 pandemic drastically changed many aspects of the traditional functioning of health systems all around the world [1]. Inevitably, the closure and reconversion of entire hospitals and outpatient services led to a reduction in the assistance guaranteed in managing chronic pathologies and in the management of usual emergencies. This applied to all sectors of medicine, including those dedicated to treating musculoskeletal pathologies [2].

As reported by the CIO (Osteosynthesis Italian Club), compared to the previous year, in Italy in 2020, there was a significant reduction in overall outpatient activities by up to 75%; it registered a reduction of up to 71% in terms of trauma that required consultation, and up to 50% regarding trauma surgery, excluding fractures of the femoral neck [3].

These data support the need for telemedicine, which represents a current challenge and can no longer be postponed in the future. Great milestones in terms of time saving, easy accessibility, cost effectiveness, hospital overcrowding reduction, and health education have been achieved in the last years in remote patient care, bridging the gap between physicians and patients that the COVID-19 pandemic caused [4]. In addition, in the orthopedic and rehabilitation field, the need to exploit the new technological means the availability to prepare remote evaluations of patients is growing [5], and it requires some methods which can be standardized and reliable in objectifying data as much as possible.

The shoulder is an anatomical area that is often affected by common musculoskeletal pathologies [6], and which, moreover, lends itself well to being remotely evaluated, albeit with a series of obvious difficulties [7].

This study aims to elaborate on a possible model for remote shoulder examination based on traditional tests and new possibilities deriving from new electronic tools to improve the quality of telemedicine in orthopedics and rehabilitation.

### Manuscript Organization

The following paragraphs show the steps through which we devised and developed our B-STEP protocol. The materials and methods section will explain the recruitment criteria for the patients on whom our protocol was tested. Next, the common in-patient objective examination and the various steps through which the telemedicine objective examination is conducted, describing each specific test in detail. In the Results section, we report the percentage completion data of the items performed based on the ASES score and the Constant score and the results of the reproducibility assessment of specific tests in telemedicine according to the observers.

## 2. Materials and Methods

We conducted an observational study between May 2020 and November 2020, at the Orthopedic and Traumatology Clinic of the Azienda Ospedaliera Universitaria Consorziale Policlinico of Bari.

We involved 10 orthopedic surgeons who are experts in the treatment of shoulder disorders; then, we asked them to elaborate on a common evaluation protocol for patients with common shoulder disease.

To study the telemedicine reproducibility of each specific test performed in a common outpatient clinical examination, we used this protocol in the follow-up of well-known patients. We recruited one patient for each of the following shoulder disorders:

1. Glenoid, humeral, and scapular fractures: a patient with proximal humerus fracture treated with plate and screws
2. Tendinitis and long head of the biceps tendon (LHB) injuries: a patient with LHB tendonitis was treated with mesotherapy
3. Rotator cuff injuries: a patient with supraspinatus (SSP) tendon lesion treated arthroscopically.
4. Acromioclavicular locations: an acromioclavicular dislocation surgically treated with a tight-rope technique
5. Glenohumeral osteoarthritis (GHO): a patient with glenohumeral osteoarthritis, not eligible for surgery, in conservative treatment
6. Shoulder instability and/or superior labrum anterior-posterior (SLAP) lesion: a patient with recurrent shoulder dislocation treated with remplissage

The recruitment criteria are shown in Table 1.

All patients signed informed consent at the time of recruitment. All procedures were performed by the principles of Helsinki.

Each of the orthopedists involved individually examined each of these 6 patients, both in person and via webcam according to the protocol previously shared.

At the end of the visits, we collected the impressions of the ten physicians separately about the possibilities and difficulties they encountered with the objective examination of the shoulder via webcam, compared with the traditional exam.

### 2.1. Outpatient Clinical Examination

**Scheme 1:**
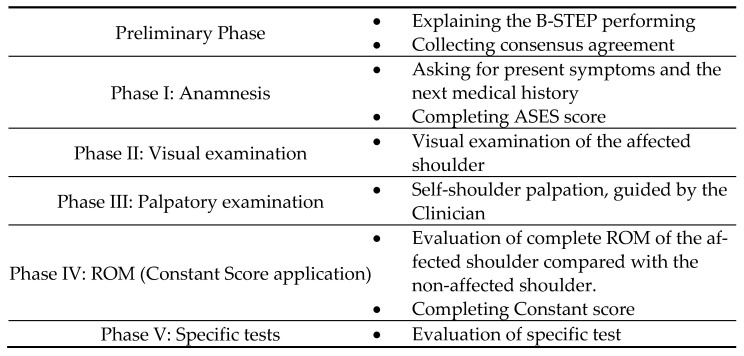
The Bari Shoulder Telemedicine Exam Protocol (B-STEP).

First, each recruited patient was examined by each physician in a live outpatient visit. We asked the observers to use the same tests present in the B-STEP in a standardized way. (See Scheme 1).

After informed consensus was collected, a comprehensive examination was performed for each patient. Medical history was noted and documentation of any surgery performed was collected. The ASES-score evaluation questionnaire was submitted to each patient.

Subsequently, an objective examination was performed, visually assessing the presence of any skin alterations or anatomical profile of the pathological shoulder, compared with the non-affected shoulder.

Next, palpation of the shoulder was performed, looking for painful areas, or alterations in subcutaneous bone profiles.

In the next phase, the entire range of motion (ROM) of both shoulders was examined, evaluating any deficit in the affected shoulder. The ROM evaluation allows for completing the items of the Constant score.

In Table 2, the description, sensitivity, and specificity, as described in the literature, of each test conducted on an outpatient basis are reported.

### 2.2. The Bari Shoulder Telemedicine Exam Protocol (B-STEP)

For the telemedicine examination (Scheme 1), we chose the smartphone app WhatsApp, as it is a widely used app within the population and easy to use, available for both smartphones and computers. These characteristics allowed us to avoid the use of additional apps or programs to be created “ad hoc” and the greater usability of the examination in telemedicine.

Selecting a commonly used clinical test, the Constant score, and a test for assessing the patient’s quality of life for shoulder disorders, such as the ASES Score, a valid protocol was developed for the most common shoulder pathologies [19,20]. The Constant score assesses subjective factors, such as the influence of pain in daily activities and quality of life, as well as objective factors such as abduction force or shoulder range of motion in intra- and extra-rotation. The ASES score evaluates the influence of pain in shoulder disorders on quality of life and daily activities from a more subjective point of view.

In addition, we selected certain specific tests for the objective examination of the shoulder that we considered highly reproducible via webcam.

This protocol was approved by each of the ten orthopedists involved in the study.

These tests were given to the patient as an interview and as reproducible evaluation maneuvers during the objective examination.

We previously collected an informed consensus from each patient.

#### 2.2.1. Preliminary Phase:

The patient is initially alerted to the possibility that the virtual visit may be recorded, ensuring the appropriate obscuration of the face and any sensitive reference that violates the privacy of the patient.

The sequence of movements and tests should be performed in front of the camera, about 1.5 m away; behind the patient, a bare wall with no reference to personal belongings is recommended.

Adequate space is needed for the patient to complete the full ROM of the shoulder. He/she should be free to move from clinostasis to orthostasis.

The patient is advised to blackout the camera if he/she must move within his/her home to respect his/her privacy.

The patient should wear a camisole that allows a complete visual examination of the affected shoulder.

The patient must be properly instructed in the performance of specific movements. Therefore, it may be helpful for the examiner himself to show these movements through the camera [21].

#### 2.2.2. Phase I: Anamnesis

Ask the patient which diseases he is suffering from. If necessary, the patient is asked to indicate with one finger the site of maximal pain. In this phase, it is possible to complete the ASES score questions.

Next, medical history is collected.

#### 2.2.3. Phase II: Visual Examination

The patient is asked to show a close-up of both shoulders to the camera to perform a visual examination as completely and accurately as possible. It is requested to show both frontally and posteriorly.

Muscle tropism is examined in comparison to the contralateral limb.

#### 2.2.4. Phase III: Palpatory Examination

The patient should perform a self-palpation of the shoulder, and asked if they experience any swelling, muscle contractures, alterations in normal anatomy, or sites of elective pain, including the LHB tendon and the acromioclavicular joint.

#### 2.2.5. Phase IV: ROM (Constant Score Application) [19]

In the Constant score, the first two items score the subjective assessment of pain and functional limitation in daily activities.

The remaining items score according to the degree of mobility of the shoulder, and the range of the respective ROM. Particularly, items 3 and 4 offer a qualitative and quantitative estimation of abduction.

The patient performs anterior flexion, abduction, and extension movements, either in frontal projection or with his back to the camera, comparing the ROM with the presumably healthy contralateral limb.

Each range of motion can be quantified on screen, to assign a specific Constant score (items 3, 5, 6).

- 0 pounds (0 kg)
- 1–3 pounds (0.45–1.3 kg); example: 1 to 3 packages of pasta in a bag
- 4–6 pounds (1.8–2.7 kg); example: a 2 L bottle up to 2 1.5 L bottles
- […]
- >24 pounds (>10 kg) example: a bale of water with 2 L bottles

Intrarotation (item 8) can be assessed with the “hand behind the back” test; for example, women can be asked to simulate the movement to “fasten their bra”, or to bring the hand behind the neck as in the act of combing their hair [22].

Extrotation (item 7) can be assessed by performing the “hand behind the neck” test with the patient placed in lateral vision, limb abducted to 90° and elbow flexed to 90°, with forearm and hand in a neutral position [23].

The patient may be asked to hold the maximum range for a few moments to capture the appropriate frame for precise range measurements using virtual goniometers.

#### 2.2.6. Phase V: Specific Tests

The first phase of ROM assessment through the CONSTANT score, as well as the patient’s compilation of the ASES score, can guide the clinician toward a diagnostic suspect. Therefore, in a normal outpatient visit, pathology-specific tests are performed. We have therefore selected some of these tests.

In the absence of direct patient contact, intra- and extra-rotation can be performed against the resistance offered by a person accompanying the patient, if present. Alternatively, the patient can perform the movements against door jambs or the wall. The test may be defined as positive if the patient reports that the movement was painful or if they feel more “weakness” than usual [23].

Subscapularis strength can be assessed with the belly press test (elbows should not move beyond the body plane). The test can be considered positive if the movement generates pain or if it reports a reduction in strength relative to the contralateral limb [24].

Empty can test (Jobe test) (SSP evaluation): shoulder abducted to 90° and in anterior flexion to approximately 45°; the upper limb is intrarotated. Normally, the patient is asked to elevate the limb further against resistance. A weight known as a standard resistance can be used [25,26].

Speed test (anterior and posterior superior cercine lesions—SLAP lesion/LHB lesions): shoulder in 90° anterior flexion. Upper limb fully extended and supinated. The patient is asked to flex the elbow against resistance, using a known weight [14,27].

To remotely evaluate shoulder instability, our observers tried to replicate the following specific tests:

Relocation test: the relocation test is a natural progression of the apprehension test and assesses relief of apprehension after manual stabilization of the shoulder. After eliciting a positive apprehension test, the examiner maintains the patient in their current position and applies a posteriorly directed force on the humeral head in an attempt to stabilize the shoulder and correct the symptoms. In a patient with anterior shoulder instability, this maneuver should bring a sub-luxed humeral head back into the correct position relative to the glenoid fossa. Resolution of guarding and apprehension suggests anterior instability and is considered a positive relocation test [19].

Apprehension test: anterior apprehension may be elicited by bringing the patient’s shoulder into a position of 90° of abduction and 90° of external rotation in either the supine or upright position. A positive exam finding is the subjective feeling of impending subluxation or dislocation when in this provocative position. It is important to note that although these symptoms may be accompanied by pain, pain itself does not produce a positive test [19].

Anterior and posterior drawer test: tests are performed with the subject lying relaxed in the supine position with the shoulder slightly flexed, neutral to 30° external rotation, and 80–120° abduction. The examiner stays to the side facing the patient, stabilizing the scapula with one hand and holding the proximal humerus with the other so that the shoulder can be kept at the intended position and rotation. Then, the proximal humerus is shifted anteriorly or posteriorly whilst applying axial force. The subtle difference between the posterior to anterior laxity examination is that the patient’s arm is flexed at 60–80°, slightly internally rotated, elbow flexed at 90°, and the humeral head shifts posteriorly during the maneuver [15].

Crank test: the crank test is performed with the patient in the upright or supine position. The shoulder is elevated 160° in the scapular plane, an axial load is applied by the examiner, and the humerus is internally and externally rotated. Pain elicited during this test, typically with external rotation, is a positive indication of a pathologic condition of the labrum. In addition, a click may or may not be felt that reproduces the patient’s symptoms of pain or catching [16,28,29,30,31].

Fulcrum test: the fulcrum test is performed with the patient supine with one shoulder on the edge of a couch. Patients felt anxiety about dislocation when the involved shoulder is externally rotated with the shoulder at 90° of abduction and the elbow at 90° of flexion in the supine position [32].

Jerk test: while stabilizing the patient’s scapula with one hand and holding the affected arm at 90° abduction and internal rotation, the examiner grasps the elbow and axially loads the humerus in a proximal direction. The arm is moved horizontally across the body. A positive result is indicated by a sudden clunk as the humeral head slides off the back of the glenoid [33].

## 3. Results

The reproducibility of the scores examined is shown in Table 3.

All the observers completed the ASES score questions via webcam (100% of the Ases score’s item).

In 37/60 cases, the observer had not completed item 4 of the Constant score, evaluating the strength of the abduction (7/8 items completed = 87.5% of Constant score).

In 4/60 cases, the observer reported an unreliable response to item 4 and item 7 (6/8 items completed = 75% of Constant score).

They reported that in performing both items, the main problem was poor patient compliance.

### Telemedicine Reproducibility of Specific Tests in the Objective Examination of the Shoulder

As shown in Table 4, there are differing opinions regarding the reproducibility of the specific tests performed in telemedicine. All orthopedic surgeons (10/10) consider the assessment of ROM reproducible, although with some limitations in the assessment of intrarotation, as evaluated above.

Regarding the evaluation of the muscular-tendinous components of the rotator cuff, 8/10 observers considered the empty can test and the speed test repeatable with good reliability. A total of 6 out of 10 observers rated the belly-press test as repeatable, whereas only 3 out of 10 observers rated the Yocum test and the Neer sign conducted via webcam as reliable.

Several unfavorable opinions were obtained regarding the evaluation of tests for instability disorders. Among the various tests examined, only the apprehension test was found to be repeatable according to 7 out of 10 observers in telemedicine.

## 4. Discussion

The COVID-19 pandemic and the need for social distancing have created challenges in healthcare access. Telemedicine has played an important role in the delivery of medical services and is likely to be of continued importance and use even after the current pandemic.

The present study aims to depict a standardized protocol to perform a reliable remote shoulder examination. Despite a small sample size of patients evaluated, with a known diagnosis, we focused on the reproducibility of specific tests commonly used in the clinical practice of an in-person objective examination.

In line with this goal, we started with shoulder evaluation scores commonly in use in orthopedic practice, such as the Constant score and ASES score [19,20].

The ASES score, a subjective test based on the evaluation of the individual’s normal daily activities and the perception of pain [34], was reproducible in all the patients examined. The Constant score was also shown to be largely reproducible during the webcam examination, and we could have a quantitative evaluation of some specific tests using everyday objects with a known weight. However, several surgeons had difficulty reproducing item 4, where the strength of abduction is quantified, and, in a few cases, there was difficulty in examining the extra rotation test.

Authors agree that most of the tests used in shoulder instability are difficult to reproduce via webcam, as these maneuvers require the in-person presence of the examiner. Such tests were excluded from our final protocol.

After performing the tests and collecting the opinions of orthopedic surgeons, we concluded that certain pathologies are more difficult to telematically assess, with likelier instability. On the other hand, many of the scores used in orthopedy can be completed with our B-STEP (e.g., Constant score, UCLA shoulder rating scale, Oxford shoulder score, ASES, DASH, and Quick DASH), allowing us to perform clinical follow-up of patients with known pathology.

The only test that could be carried out on the patient by telemedicine is the anterior apprehension test, which consists of abduction of the shoulder to 90° and simultaneous external rotation to 90°. This test has the aim of reproducing the patient’s fear or feeling of a possible anterior dislocation of the shoulder, in which case the test is considered positive [15,16,19].

Given the association between joint laxity and AMBRI-type shoulder instability (atraumatic, multidirectional, bilateral), it is useful to assess the patient’s ligamentous laxity according to the Beighton scale [35,36,37,38].

The most-used scale in the clinical workup of joint laxity is the Beighton nine-point scale. The scale includes only common symptoms: (1) extension of the MCF in the 5th finger beyond 90 degrees, (2) abduction of the thumb on the forearm, (3) hyperextension of the elbow by more than 10 degrees, (4) hyperextension of the knee by more than 10 degrees, and (5) contact of the palm of the hands on the floor with the lower limbs extended. One point is given for each finding. A score of ≥4 points is used to diagnose joint laxity [36].

The evaluation of the Beighton scale combined with medical history and signs of shoulder instability may indicate the need for further investigation, which cannot be assessed by a telemedicine visit.

Other recent studies have shown that in-person clinical examination is necessary to confirm suspected instability of the shoulder [39,40,41,42,43,44].

Our protocol appears to be in line with other methodologies used in other studies [40]. However, the use of everyday objects is promoted in our study to be able to quantify functional shoulder deficits and to standardize patient assessment and follow-up as much as possible.

## 5. Conclusions

The B-STEP is a useful protocol for the standardization of the objective examination of the shoulder via webcam. The purpose of this protocol is to perform the follow-up on post-treatment recovery, monitor the evolution of the pathology in patients conservatively treated, and reduce waiting lists for outpatient visits.

Further studies—blinded, or with a larger cohort of patients—are necessary to determine if the B-STEP protocol can diagnose pathology in unknown patients, evaluating its sensitivity and specificity for each pathology.

The telematics approach can also be useful in the online assessment of patient follow-up imaging, complementing the clinical assessment of the B-STEP.

## Figures and Tables

**Table 1:** Inclusion and exclusion criteria.

Inclusion Criteria
Over 18 years of age
Capable of expressing informed agreement
A known diagnosis
At least 6 months follow up
Availability of electronic devices with webcam (cell phones, tablets, pc)
**Exclusion Criteria**
The concomitance of more than shoulder pathology
Presence of other rheumatological, orthopedic, or neurological ipsilateral upper limb pathologies
Inability/unwillingness to be remotely monitored.

**Table 2:** Sensitivity and specificity of commonly used specific tests.

Specific Test	Tested Pathology	Test Description	Criteria for Positivity	Sens/Spec in Literature
	**Rotator cuff integrity**			
Intra-rotation against resistance [8]	Subscapularis	Upper limb along the trunk, forearm flexed 90° in a neutral position. Intrarotation is forced against a door jamb	Test positive if evoking pain or strength deficit relative to contralateral is reported	Sens: 0.56 Spec: 0.87
Hand behind the back [9]	Subscapularis	Shoulder intrarotation bringing the hand behind the back (“bra lacing movement”)	Range of motion assessment (hand at lumbar level, hand at thoracolumbar level, …)	Reliability: Weighted k 0.73
Belly-press [10]	Subscapularis	The upper limb was slightly abducted and intrarotated by bringing the palm up to the navel level. The patient is asked to intrarotate the limb by pressing the hand against the abdomen	Positive test if the patient fails to keep the elbow in the frontal plane during the intrarotation movement	Sens: 0.40 Spec: 0.98
Extra-rotation against resistance [11]	Infraspinatus/teres minor	Upper limb along the trunk, forearm flexed 90° in a neutral position. The patient is asked to force extrotation against a door jamb	Test positive if evoking pain or strength deficit relative to contralateral is reported	Sens: 0.46 Spec: 0.94
Extrotation with abducted arm [11]	Infraspinatus/teres minor	Abduction of the shoulder to 90° and simultaneous external rotation.	The feeling of apprehension or pain at maximum degrees of extrotation	Sens: 0.40–0.62 Spec: 0.42–0.87
Hand behind neck [9,12]	Infraspinatus/teres minor	The patient brings the hand behind the neck	ROM amplitude is assessed: hand on the neck, hand on the ipsilateral scapula, and hand on the opposite scapula. The test is positive if it evokes pain	Reliability: Weighted k Intra-examinator: 0.80 Inter-examinator: 0.90
Jobe test [13]	SSP	Shoulder abducted to 90°, anterior flexion to approximately 45°; a supra-rotated limb. The patient is asked to further elevate the limb against known resistance	Test positive if evoking pain or strength deficit relative to contralateral is reported	Sens: 0.50–0.69 Spec: 0.62–0.87
	**SLAP lesions/LHB**			
Speed test [14]	SLAP lesion/long head bicep integrity	Anterior humerus flexion at 90°, upper extremity fully extended and supinated. The patient is asked to flex the elbow against resistance using a known weight	Test considered positive if it evokes pain at the level of the groove of the long head of the biceps	Sens: 0.20–0.60 Spec: 0.38–0.78
	**Impingement Syndrome**			
Painful ROM arch in abduction [14]	Impingement syndrome/subacromial bursitis	Abduction of the upper limb up to about 90°; possible extrotation of the limb to complete the ROM	Positive test if evokes pain	Sens: 0.56 Spec: 0.76
	**Shoulder instability**			
Anterior apprehension test [15,16]		Abduction of the shoulder to 90° and simultaneous external rotation.	The feeling of apprehension or pain at maximum degrees of extrotation	Sens: 0.40–0.62 Spec: 0.42–0.87
Relocation test [17]		Supine patient; glenohumeral joint at the edge of the table. Arm in 90° abduction and fully extrotated, with the elbow flexed 90°. A direct posterior force is applied to the humeral head	The test is positive if the force application reduces pain or apprehension	Sens: 0.81 Spec: 0.92
Drawer test [17]		Supine patient; glenohumeral joint at the edge of the table. Arm at 60–80° of abduction and in neutral rotation. A force is applied to translate the humeral head anteriorly	Test positive if eliciting pain	Sens: 0.28–0.53 Spec: 0.71–0.85
Crank test [14]		Patient supine; arm elevated to 160°. An axial force is applied to the humerus while the shoulder is intra- and extra-rotated.	Test positive if eliciting pain	Sens: 0.34 Spec: 0.75
Fulcrum test [15]		Patient supine; arm abducted to 90° and elbow flexed to 90°; an axial force in compression is applied to the humerus and the shoulder is rotated.	Test positive if eliciting pain	Sens: 0.61 Spec: 0.54
Jerk test [18]		Patient seated. The arm intrarotated and elevated to 90° of abduction. Then, an abduction movement is performed on the horizontal plane and an axial force is applied to the humerus.	Test positive if eliciting pain	Sens: 0.73 Spec: 0.98

**Table 3:** Reproducibility of Constant score and ASES score, according to the observation of Orthopedic Surgeons, during the examination in TELEMEDICINE.

	Proximal Humeral Fracture	LHB Tendinitis	SSP Lesion, Surgically Treated	Acromion-Clavear Luxation	Hemarthrosis	Shoulder Instability
Orthopedic physician 1	Constant: 87.5% ASES: 100%	Constant: 100% ASES: 100%	Constant: 87.5% ASES: 100%	Constant: 100% ASES: 100%	Constant: 87.5% ASES: 100%	Constant: 75% ASES: 100%
Orthopedic physician 2	Constant: 100% ASES: 100%	Constant: 100% ASES: 100%	Constant: 100% ASES: 100%	Constant: 100% ASES: 100%	Constant: 100% ASES: 100%	Constant: 100% ASES: 100%
Orthopedic physician 3	Constant: 100% ASES: 100%	Constant: 87.5% ASES: 100%	Constant: 87.5% ASES: 100%	Constant: 87.5% ASES: 100%	Constant: 87.5% ASES: 100%	Constant: 87.5% ASES: 100%
Orthopedic physician 4	Constant: 87.5% ASES: 100%	Constant: 87.5% ASES: 100%	Constant: 75% ASES: 100%	Constant: 87.5% ASES: 100%	Constant: 87.5% ASES: 100%	Constant: 87.5% ASES: 100%
Orthopedic physician 5	Constant: 100% ASES: 100%	Constant: 87.5% ASES: 100%	Constant: 87.5% ASES: 100%	Constant: 100% ASES: 100%	Constant: 87.5% ASES: 100%	Constant: 87.5% ASES: 100%:
Orthopedic physician 6	Constant: 100% ASES: 100%:	Constant: 75% ASES: 100%:	Constant: 87.5% ASES: 100%:	Constant: 87.5% ASES: 100%:	Constant: 100% ASES: 100%:	Constant: 87.5% ASES: 100%:
Orthopedic physician 7	Constant: 87.5% ASES: 100%:	Constant: 87.5% ASES: 100%:	Constant: 87.5% ASES: 100%:	Constant: 87.5% ASES: 100%:	Constant: 87.5% ASES: 100%	Constant: 87.5% ASES: 100%
Orthopedic physician 8	Constant: 100% ASES: 100%:	Constant: 87.5% ASES: 100%	Constant: 100% ASES: 100%	Constant: 75% ASES: 100%	Constant: 87.5% ASES: 100%	Constant: 87.5% ASES: 100%
Orthopedic physician 9	Constant: 87.5% ASES: 100%	Constant: 87.5% ASES: 100%	Constant: 87.5% ASES: 100%	Constant: 87.5% ASES: 100%	Constant: 100% ASES: 100%	Constant: 100% ASES: 100%
Orthopedic physician 10	Constant: 87.5% ASES: 100%	Constant: 87.5% ASES: 100%	Constant: 100% ASES: 100%	Constant: 87.5% ASES: 100%	Constant: 100% ASES: 100%	Constant: 87.5% ASES: 100%

**Table 4:** Reproducibility assessment of specific tests in telemedicine according to the 10 observers.

Tab 3	ROM	Bellypress	Empty-Can Test	Speed Test	Yocum	Neer Sign	Relocation Test	Apprehension Test	Drawer Test	Crank Test	Fulcrum Test	Jerk Test
Orthopedic physician 1	Yes	Yes	Yes	Yes	No	Yes	No	Yes	No	No	No	No
Orthopedic physician 2	Yes	Yes	Yes	Yes	No	No	No	Yes	No	No	No	No
Orthopedic physician 3	Yes	No	Yes	No	Yes	No	No	Yes	No	No	No	No
Orthopedic physician 4	Yes	No	Yes	Yes	No	Yes	No	No	No	No	No	No
Orthopedic physician 5	Yes	Yes	No	Yes	No	Yes	No	Yes	No	No	No	No
Orthopedic physician 6	Yes	Yes	No	Yes	No	No	No	Yes	No	No	No	No
Orthopedic physician 7	Yes	Yes	Yes	Yes	Yes	No	No	Yes	No	No	No	No
Orthopedic physician 8	Yes	No	Yes	Yes	No	No	No	No	No	No	No	No
Orthopedic physician 9	Yes	Yes	Yes	Yes	No	No	No	Yes	No	No	No	No
Orthopedic physician 10	Yes	No	Yes	No	No	No	No	No	No	No	No	No
Reprod.:	10/10	6/10	8/10	8/10	3/10	3/10	0/10	7/10	0/10	0/10	0/10	0/10

## Data Availability

The data that support the findings of this study are available from the corresponding author upon reasonable request.
